# On including assessments in the calculation of teaching loads

**DOI:** 10.3205/zma001658

**Published:** 2024-02-15

**Authors:** Volkhard Fischer

**Affiliations:** 1 Medizinische Hochschule Hannover, Studiendekanat Bereich Evaluation & Kapazität, Hannover, Germany

**Keywords:** assessments, teaching load, examination parameters, curricular standard value

## Abstract

Next to courses and seminars, tests and assessments represent the main parameters with which to describe an academic study program independent of its curricular content. Thus, the quality of education depends not only on the quality of the courses taught and how they are interconnected, but also on the quality of testing and the feedback given to students regarding their performance.

Course quality should be ensured through course evaluation. The economic cost of courses is calculated based on the required teaching load. The concept of teaching load stems from the time when program planning was instructor-centered. The main variable in the rules and regulations governing university study was the number of hours per week per semester (or number of course hours). But even in today's student-centered planning, which uses ECTS credits per module as the variable, teaching loads are still used to determine the number of staff necessary to offer an academic study program.

Some universities also include the assessments in the evaluation. Yet the economic costs of testing are de facto ignored almost everywhere, and this does not bode well for the quality of the assessments. Much progress would be made to improve higher education if assessments counted as part of the teaching loads and the curricular norm values.

This paper identifies which requirements must be considered in order to include assessments in teaching loads.

## Abstract (de)

Neben Lehrveranstaltungen stellen Prüfungen die zentralen Bestimmungsgrößen zur Beschreibung eines Studiengangs unabhängig von seinen Inhalten dar. So hängt die Ausbildungsqualität einerseits von der Qualität der Lehrveranstaltungen und ihrer Verzahnung untereinander, andererseits aber auch von der Qualität der Prüfungen und der mit ihnen erfolgenden Leistungsrückmeldungen an die Studierenden ab.

Die Qualität der Lehrveranstaltungen soll durch deren Evaluation sichergestellt werden. Der ökonomische Aufwand für die Lehrveranstaltungen wird über das benötigte Lehrdeputat ermittelt. Lehrdeputate stammen aus einer Zeit als dozierendenzentriert akademische Lehre geplant wurde. Zentrale Kenngröße in den Studienordnungen waren die Semesterwochenstunden (bzw. Lehrveranstaltungsstunden). Aber auch in der aktuellen studierendenzentrierten Studiengangsplanung mit ECTS-Punkten pro Modul als Kenngröße, haben Lehrdeputate ihren Nutzen zur Bestimmung des für die Studiengangsdurchführung notwendigen Personalbedarfs nicht verloren.

An manchen Standorten gehen die Prüfungen auch mit in die Evaluation ein. Der mit Prüfungen verbundene ökonomische Aufwand wird de facto aber fast überall ignoriert. Für die Qualität von Prüfungen verheißt dies nichts Gutes. Würde man die Prüfungen in die Berechnung der Lehrdeputate und der Curricularnormwerte einbeziehen, wäre für die Verbesserung der studentischen Ausbildung schon viel gewonnen.

Welche Voraussetzungen beachtet werden müssen, um Prüfungen in die Erfassung der Lehrdeputate mit einzubeziehen, behandelt dieser Artikel.

## Introduction

Assessments are an important component of university study programs and can lead to long legal battles. Two rulings handed down by the Federal Administrative Court (Bundesverwaltungsgericht) [1], [2] have resulted in a more precise wording of the relevant passages in the regulations governing the study and testing of midwifery* (Studien- und Prüfungsverordnung für Hebammen)* [https://www.gesetze-im-internet.de/hebstprv/BJNR003900020.html], although these rulings referred to other courses of study in Germany. Precisely because these court decisions address the basic framework for objective testing, it is surprising that such improvements and revisions are still needed. It is not clear why this is the case since this issue is clearly a topic of wide discussion: There are

- international standards for tests [3], [4], [5], [6],
- recommendations for practitioners [7], [8], and
- literature on the laws governing academic testing ([9] or a new edition, [10]).

On the other hand, tests and assessments are traditionally part of the pre- and post-work done for courses. And only the courses themselves are factored in to the calculation of curricular norm values (*Curricularnormwerte* or CNW) and counted toward the individual teaching load (or teaching duties). If it is understood that a university study program is comprised of (classroom-based and/or online) courses, different types of feedback, self-guided study and extra-curricular activities, it is clear that feedback, together with the courses, belongs to the responsibilities owed by the university and its teachers to the students.

Several state-level (Land) rules and regulations regarding the assignment of teaching duties in higher education (*Lehrverpflichtungsverordnung*, or LVVO) contain statements phrased to allow faculties the option of paying tests and assessments the attention due by treating them as a subgroup of the feedback students receive regarding their academic progress. Passages from four LVVOs are presented in table 1 (Tab. 1) as examples.

The requirements which should be met so that a university faculty can take advantage of this option are covered in the following. The background for this entails the following considerations:

- P1) Assessments should be sufficiently defined so that the resulting interference with students’ rights is still legally compliant.
- P2) Assessments do not belong to conventional seminars and courses and generally are not used in the calculation of teaching load, but rather count as part of the work done before and after teaching courses.
- P3) If an LVVO does not permit the inclusion of courses not originally provided for, then these courses must be calculated toward the teaching load in a manner similar to conventional courses.
- S) Thus, in order to include assessments in the teaching load, it is necessary to establish which of the assessment features that must be sufficiently defined to ensure specificity correspond to the factors used to calculate teaching load and if these features can be considered separately from the course.

In its decision regarding a digitally administered module assessment, the Administrative Court in Hannover formulated a list of requirements for the Hannover Medical School that its examination regulations should satisfy [11]. Even if the judicial system is now more familiar with assessments using electronic devices [10], the requirements stipulated by the court at that time (see table 2 (Tab. 2)) represent not only important guidance to ensure the legality of the tests and assessments administered over the course of an academic study program, but also for including them in the calculation of teaching loads. The starting point for the legal considerations is that the basic right to freely choose an occupation, as provided for in § 12 (1) of the Basic Law of the Federal Republic of Germany (Grundgesetz), is restricted since a final failure on an assessment permanently hinders future professional practice. Even in the very general case of a test or assessment, the materiality principle must be satisfied, and this fundamental right may only be restricted on legal grounds or within the scope granted by law under a statute. To comply with this, assessment procedures and requirements should be based on the principle of equal opportunity and it should be ensured, to the greatest extent possible, that the methods for grading assessments will stand up to judicial review. This aligns very well with the international standards for objective, valid and reliable assessments [3], [4], [6].

The first column of table 2 (Tab. 2) specifies the variables used to calculate the curricular percentage (CAp) of a conventional course. The second column lists features that need to be sufficiently defined for a test, according to the court decisions cited above. It does not, however, present an exhaustive list. All of the components of these features are then listed in the third column. The variables needed to include assessments in the teaching load are what enables quantification analogous to that for the conventional courses.

The features listed in the second column of table 2 (Tab. 2) all affect the objectivity of assessments, whereby it is already clear that they are in no way sufficient to ensure “good” tests. Nonetheless, it is (usually) the objectivity of an assessment that is the subject of legal scrutiny. Exceptions confirm this rule, such as the judgment handed down in the “numerus clausus case” by the Federal Constitutional Court (Bundesverfassungsgericht) [12]. For, at the time, when clarifying the constitutionality of multiple-choice testing, the fundamental question was not about the reliability of state medical exams using the multiple-choice format, but rather about their level of difficulty across different cycles.

Understood metaphorically, this means that the rules and regulations required by law affect, above all, the foundation, not the walls nor the roof, of the structure “housing” the tests and examinations connected with a particular study program and that they should be self-evident. For this reason, they are referred to, for instance, in the “recommendations” [8]. The focus of the recommendations is based, however, primarily on the threats to the validity and reliability of assessments because it is thought that the greatest number of weak points can be found there. To this extent, the characteristics in the second column of table 2 (Tab. 2) only represent a partial list of the criteria for “good” or legally compatible assessment, but they do make it feasible to fulfill the demand contained in table 1 (Tab. 1) to define the other courses relevant to computing teaching load.

## Assessments

In its examination regulations, the Medical University of Vienna differentiates in a very helpful manner between courses in which ongoing assessment is an intrinsic part, course-based tests, and comprehensive assessments [13]. There is also, in addition to the bachelor thesis, a final exam at the end of the study program. These differences clearly show the range that assessments can have and why it is not unreasonable to consider separately counting at least certain forms of testing toward the teaching load. The order in which these assessment formats are addressed in the following is based according to the severity of the consequences generally associated with them [14].

The question as to whether or not feedback is graded is only of secondary importance here. What is relevant is if the feedback brings consequences with it; if the assessment can only be repeated so many times if it has not been passed. Therefore, as a first step there needs to be a delineation between feedback with no consequences (course attendance certificates, formative assessments) and feedback with consequences (summative assessments). This would mean that much of the ungraded feedback designated in the study and examination regulations as academic coursework is summative assessment because failing interferes with the free choice of occupation by extending the length of study time or even leading to disenrollment from the study program.

### Student theses and state exams

Regulations governing academic study define the different types of courses offered in a study program. In them there is usually also at least one section on the requirements for approval and completion of a student thesis. In regard to the inclusion of student theses in teaching loads, separate sections were added to the LVVOs only after the Bologna Process (see table 3 (Tab. 3)), although student theses are much older than these reforms.

This makes it clear that this traditional assessment format can be counted toward the teaching load. The difference here is that in the case of thesis advising, the number of hours spent advising cannot be determined objectively, as is the case with conventional classroom teaching, but rather only indirectly based on the student workload.

The state exams in dentistry and medicine are not currently included in the calculation of the curricular norm values for these study programs. No teaching effort is assigned at all to these exams in the overall calculation of the curricular norm value for each program. This is surprising, considering that, for example, in the dental licensing regulations (*ZApprO*) the definitions of the different sections of the state exam go on for 22 pages [https://www.gesetze-im-internet.de/zappro/BJNR093310019.html] and a total of 12 pages are needed in the current version of the medical licensing regulations (*ÄApprO*) [https://www.gesetze-im-internet.de/_appro_2002/BJNR240500002.html]. This is surprising because these characteristics – mandated by the administrative courts precisely for these state exams to ensure sufficient definition of a test–are duly defined in the relevant university rules and regulations, and they are normally documented so well when administering assessments that it would be thoroughly feasible to include this in the calculation of an individual teaching load and the curricular norm value.

In contrast to bachelor and master theses, the institution responsible for assessment is not the university but a state-level examination office. Carrying out the actual assessment, however, lies with the university faculty members. Thus, factoring the assessment times into teaching loads would be entirely justified; the more so as no one doubts that the oral assessment formats, unlike the written state exams consisting of nationally standardized questions, require unique preparation by teachers at specific universities. Reversely, the administrative courts do not recognize the administering of these state exams as a reason to reduce teaching load because they are clearly part of the academic teaching duties.

### Comprehensive assessments

Comprehensive assessments cover the content of all courses offered in a semester or academic year, regardless if the comprehensive assessment is designed to be formative or summative [13]. Like the final exams at the end of a study program or the state exams, summative comprehensive assessments are in no way conceived of as preparatory or follow-up work for specific courses. It is very difficult to view creating, administering and grading them as part of the work done before and after a course or module. Nonetheless, they are an integral component of the study program, for instance, when they determine a student's progression to the next phase of study.

A current example for such a summative comprehensive assessment is found in the Objective Clinical Examination (OSCE) that is required under § 37 of the revised draft legislation for medical licensing (ÄApprO) [15]. According to § 37 of the revised licensing regulations, the future OSCE would meet all of the requirements laid out by the LVVOs to count it as part of the teaching load. Also, the duration of the assessment time would even be set down in statute.

One example of a formative comprehensive assessment is voluntarily taking the progress tests offered at many universities [16] if they are irrelevant to a student's passage on to the next level. These types of assessments represent a special form of self-guided study.

### Module tests

Course-specific tests come at the end of a course concluding it in the form of a singular occasion on one day, or at a maximum on two. The Viennese regulations [13] leaves open whether these cover only the block's lectures or a combination of lecture, seminar and practicum, as would be assumed for a module test. In general, it is module tests that are meant here.

At first glance, there are many reasons to continue viewing the assessments that give students feedback on their academic performance at the end of a module as simply part of the pre- and post-work associated with teaching. This traditional approach also makes sense for unstandardized assessments of unclear objectivity which do not (and are not meant to) fulfill the minimum requirements for documented assessment. However, as a result, they are on par with a testing practice that has been repeatedly rejected by the administrative courts as unlawful [8], [9].

Since modules are often a combination of different teaching and course formats, it is impossible to clearly assign module testing to the preparatory or follow-up work done to teach a particular course. Moreover, a standardized test requires a greater level of effort if it is to be objective, reliable and valid. A written multiple-choice test – to which the administrative courts, in a misjudgment of the literature on assessment quality [17], generally show a particular distrust – with 60 or more questions cannot be created in the same amount of time needed to write a two-hour lecture. Furthermore, a 12-station OSCE also requires more time and effort than teaching a cohort of medical students in small groups in the SkillsLab for four hours each [18].

In addition to the effort required for assessments based on table 2 (Tab. 2), purely pragmatic reasons also speak for inclusion in the teaching load. It does not take special genius to list the lecture and the assessment separately in a module description, to specify the testing format in the examination regulations, or to list explicitly all of the module tests. Even if the lecture itself should remain unchanged for several years, it would be possible, over the same course of time, to change the written test with open-ended questions into a multiple-choice test taken on electronic end devices, and this can then be documented by means of a minor revision to the examination regulations.

In regard to differentiating between module tests with and without consequences, the same applies as for the final exams taken at the end of a study program: formative module tests pose a special form of self-guided study.

### Courses with inherent examination character

If grades relevant to passing are given during a course, then, according to the Vienna Medical University’s examination regulations [13], this involves a course with ongoing assessment as an inherent part. The model examination regulations for the Leibniz University in Hannover [19] mentions course-related assessments. Both terms express the fact that this does not involve one test occasion, but rather a repetition of several occasions of assessment that are then evaluated as an overall grade.

Classic examples of such graded work are seminar presentations, making plaster casts and models in dental medicine, and writing lab reports for practicums in the natural sciences. These student assignments cannot be separated from the classroom sessions precisely because they are completed during the normal weekly seminar or practicum hours. They also cannot be counted separately in the teaching load as assessments, since an individual student’s presentation during a seminar session is coursework for the listeners and a test of ability for the presenter, whereby the roles are switched from session to session. Furthermore, in the student-to-teacher ratio, “student” refers to both the presenter (examinee) and the listeners.

The case is different for a seminar in which different topics are covered during the sessions and which are then gone into more depth in seminar papers that are turned in at the end of the semester. A practicum in which clinical examination techniques are practiced is not a course with ongoing inherent examinations if the summative feedback is given via an OSCE at the very end. Because these assessments are chronologically separate from the course, they have a unique character and fall into the category described in the previous section.

## Discussion

If feedback is meant to give students a differentiated evaluation of their learning progress, then how it is designed, delivered and evaluated entails time and effort. The justification given by the German Association of University Professors and Lecturers (Deutsche Hochschulverband) for its push in 2019 to reduce the teaching load of university teachers is based, among other things, on the increased requirements placed on assessments [20]. However, such a solution does not promote the creation of high-quality assessments, but rather is just less encumbering than the existing process. Moreover, since the feedback from summative assessments is connected with consequences for a student's future career path, this feedback should be sufficiently defined so that it is not only objective, valid and reliable [21], but also compatible with the law. The higher an assessment's stakes are, the more transparent the conditions under which it is taken should be and the more detailed (legally speaking) the assessment's specification should be [9]. The proposal made in this paper to count assessments separately as part of the teaching load would work to facilitate this.

Only the relevant examination regulations containing explicit definitions according to the characteristics listed in table 2 (Tab. 2) can, as the lowest bar, apply to all of the summative assessment formats described in the previous section. Due to their lack of consequences, formative assessments are not required to be explicitly defined or specified in the examination regulations for reasons of legal conformity. Meeting this requirement, however, would be necessary in order to count this type of assessment toward the teaching load, as well as to improve the quality of higher education overall.

**Figure 1:**
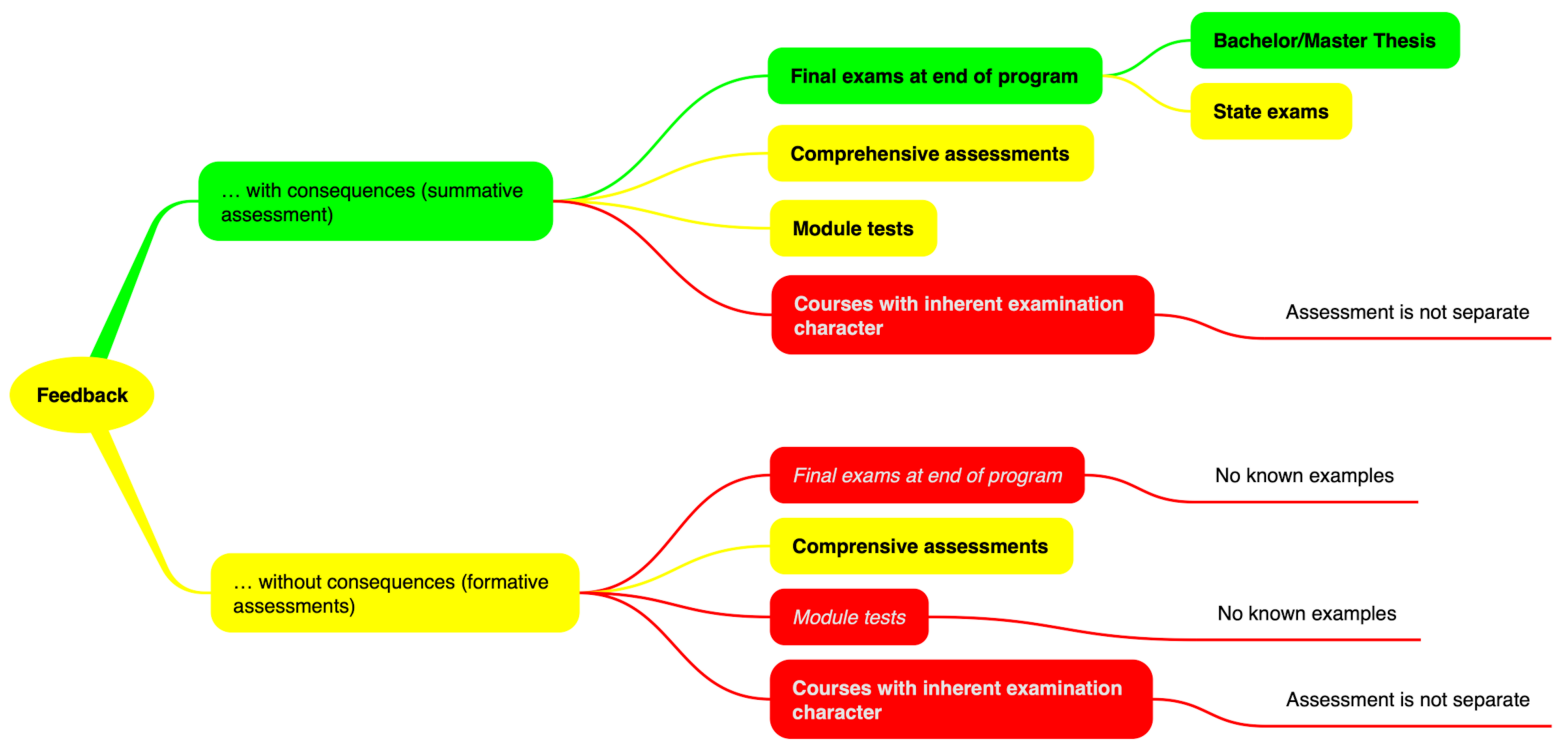
Delineation between feedback which is already counted in teaching loads (green), which could potentially count toward teaching loads (yellow), and feedback that cannot be included in the teaching load (red).

Figure 1 (Fig. 1) illustrates the delineations suggested in this paper between what is already counted toward teaching load (green), what could potentially be counted (yellow), and which assessments cannot be counted (red). If this classification is deemed acceptable, the question then arises as to why the criteria viewed by the administrative courts as necessary to sufficiently define assessments are, in turn, necessary only in part when calculating the teaching load.

To factor a course into the teaching load, its topic, duration in hours, and the standard teacher-to-student ratio or group size must be known. It is generally assumed that a teacher is constantly present in the classroom. These variables are also defined for assessments in legally compliant examination regulations and are indispensable for inclusion in the teaching load. The fact that there are still many study and examination regulations which do not implement this only says something about the affected study programs’ lack of transparency.

Under which conditions students are admitted to take a course or sit for an exam, how course attendance or passing is certified, what the consequence are for not attending or failing a course, and how all of this is documented are usually explicitly laid down in good study and examination regulations. Yet this information is irrelevant for both courses and assessments when calculating their curricular percentage (CAp).

The question remains as to why the basic standards specified by the Federal Constitutional Court for legally sound assessments are not explicitly implemented in all examination regulations, especially since these are also basic criteria for assessment objectivity. The answer may have to do with the unwillingness to depart from long-established practices and the accounting for costs [14]. However, the cost for one individual assessment generally does not change significantly if the test is based on international standards [3], [22] and/or its design is theory-based [23]. Perhaps the working hours expended would change if the assessments could be counted as part of the teaching load. However, working hours already now involve preparing, giving and evaluating assessments. What would definitely see an increase are the estimated costs of a study program or its curricular norm value. Whether the actual cost increases depends on the present quality of precisely these assessments (or the effort invested in them). But if the notion is taken seriously that assessments could constitute an interference with an individual's basic rights, then they should not be hidden in the budget under “miscellaneous”. Perhaps then, the absolute number of graduates will increase.

## Author’s ORCID

Volkhard Fischer: 0000-0001-8499-9437

## Competing interests

The author declares that he has no competing interests.

## Figures and Tables

**Table 1:**
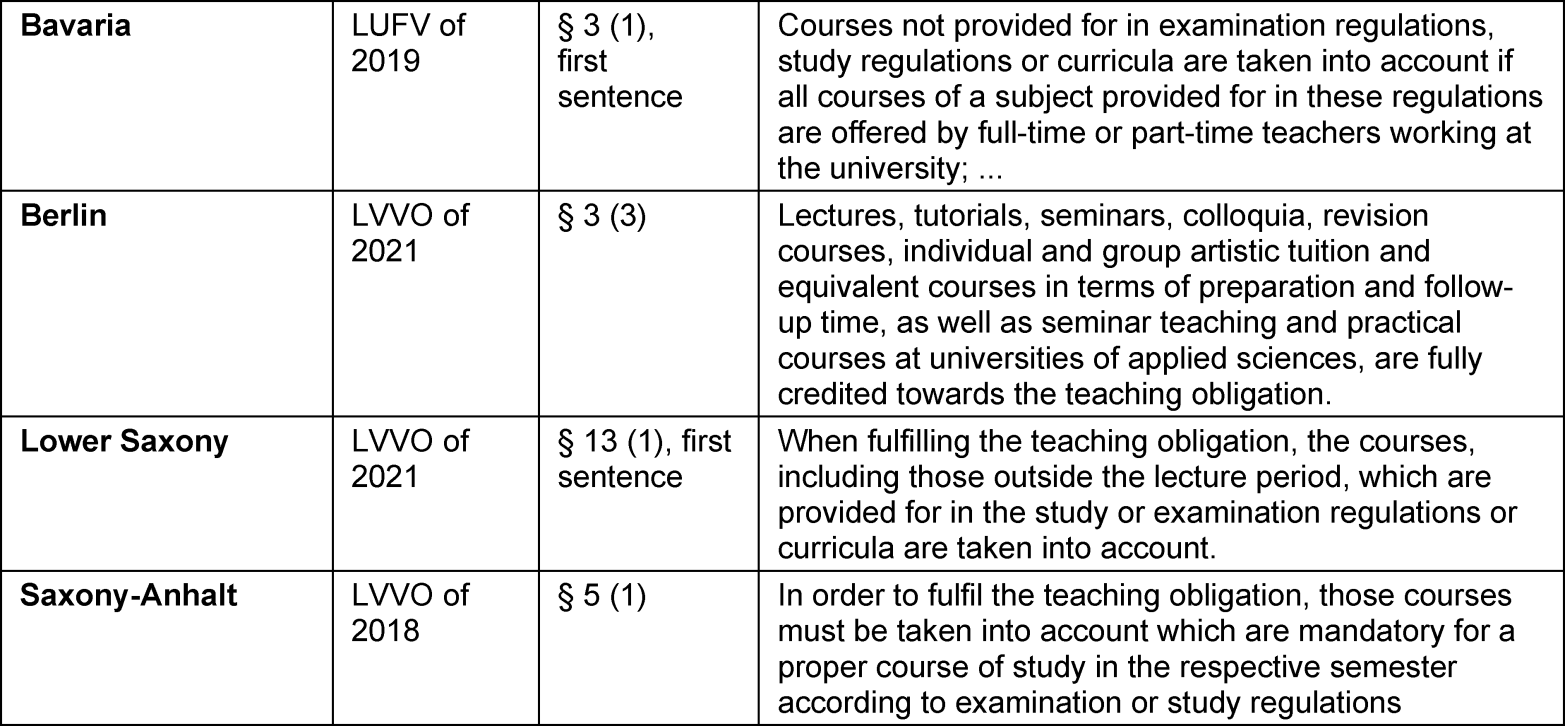
Excerpts from different state regulations governing the assignment of university teaching duties that allow the possibility of counting courses or lectures towards the calculation of the teaching load (translated)

**Table 2:**
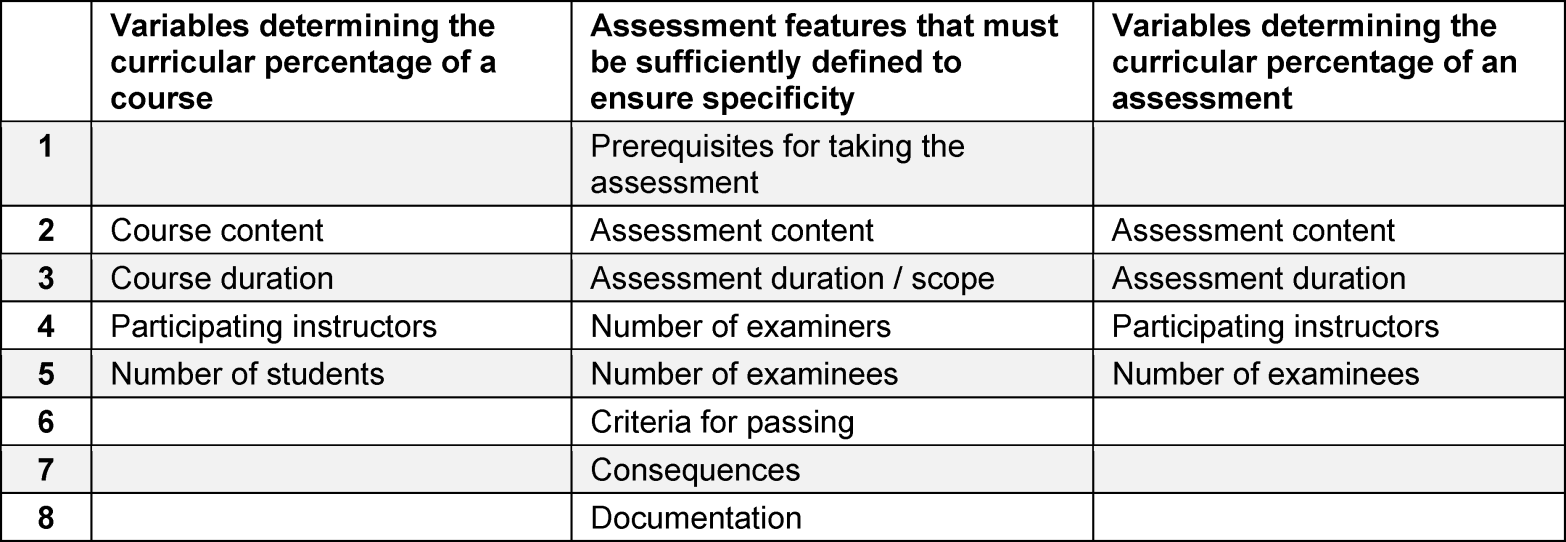
Necessary features of an assessment, as defined in the examination regulations, to ascertain sufficient specificity, to include it in the calculation of teaching load, and to determine its curricular percentage

**Table 3:**
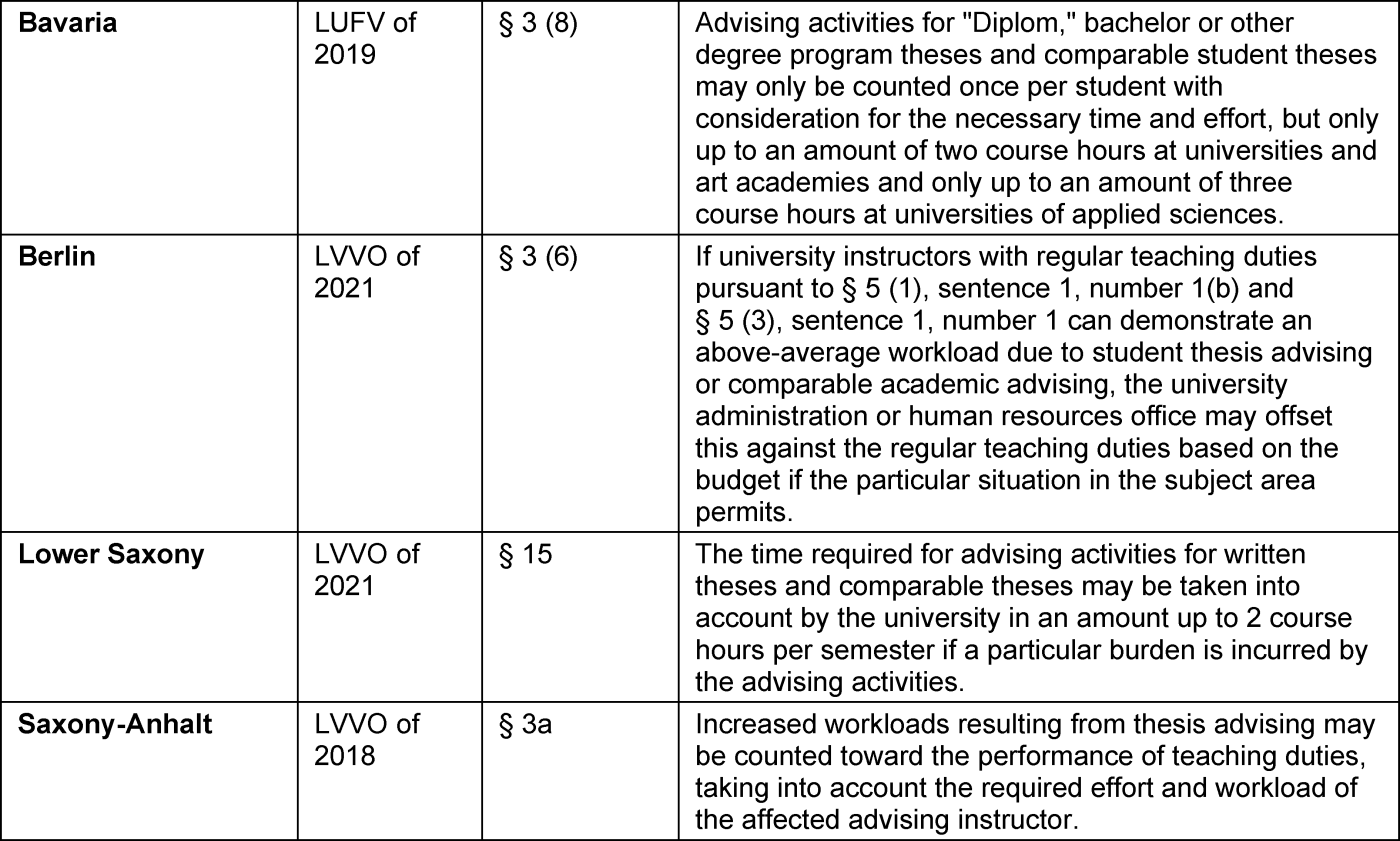
Excerpts from different regulations governing teaching duties on the inclusion of student theses in the calculation of teaching load (translated)
